# Accuracy of dental implant placement with computer-guided surgery: a retrospective cohort study

**DOI:** 10.1186/s12903-022-02046-z

**Published:** 2022-01-16

**Authors:** Jeong-Kui Ku, Junggon Lee, Hyo-Jung Lee, Pil-Young Yun, Young-Kyun Kim

**Affiliations:** 1 Department of Oral and Maxillofacial Surgery, Gangnam Severance Hospital, Yonsei University College of Dentistry, 211, Eonju-ro, Gangnam-gu, Seoul, 06273 Korea; 2 Department of Oral and Maxillofacial Surgery, Section of Dentistry, Seoul National University Bundang Hospital, 82 Gumi-ro, 173 Beon-gil, Bundang-gu, Seongnam, 13620 Korea; 3 Department of Periodontology, Section of Dentistry, Seoul National University Bundang Hospital, 82 Gumi-ro, 173 Beon-gil, Bundang-gu, Seongnam, 13620 Korea; 4 Department of Dentistry and Dental Research Institute, School of Dentistry, Seoul National University, 101, Daehak-ro Jongno-gu, Seoul, 03080 Korea

**Keywords:** Accuracy, Computer-guided surgery, Dental implant, Implant-guided surgery, Virtual plan

## Abstract

**Background:**

The aim of this study was to assess the accuracy of virtual planning of computer-guided surgery based on the actual outcomes of clinical dental implant placement.

**Methods:**

This retrospective study enrolled patients among whom implant treatment was planned using computer-guided surgery with cone beam computed tomography (CBCT). The patients who received implant according to the guide with the flapless and flapped approach were classified as group 1 and 2, respectively, and the others who could not be placed according to the guide were allocated to the drop-out group. The accuracy of implant placement was evaluated with the superimposition of CBCT.

**Results:**

We analyzed differences in the deviated distance of the entrance point and deviated angulation of the insertion of implant fixtures. With regard to the surgical approach, group 2 exhibited greater accuracy compared to group 1 in deviation distance (2.22 ± 0.88 and 3.18 ± 0.89 mm, respectively, *P* < 0.001) and angulation (4.27 ± 2.30 and 6.82 ± 2.71°, respectively, *P* = 0.001). The limitations of guided surgery were discussed while considering the findings from the drop-out group.

**Conclusions:**

Computer-guided surgery demonstrates greater accuracy in implant placement with the flapless approach. Further research should be conducted to enhance the availability of guides for cases with unfavorable residual bone conditions.

## Background

The combination of virtual engineering with the digitization of information in dentistry has given rise to a new and innovative direction for dental diagnosis and treatment. In particular, computer-based implant-guided surgery has been developed to overcome the limitations associated with traditional surgical plans and has significantly improved the accuracy of implantation and allowed for minimally invasive surgery [1, 2]. Computer-based implant-guided surgery can plan the optical implant position based on three-dimensional evaluation of the surrounding vital anatomic structures and future prosthetic requirements [2].

For implant placement, the conventional flap approach involves the exposure of the residual bone using a full-thickness mucoperiosteal flap followed by implant placement and primary closure [3]. Without flap elevation, a flapless approach for implant placement has several advantages such as being minimally invasive, being less painful, enhancing blood circulation, and preventing periosteum damage [4–8]. These advantages have a synergic effect with computer-guided surgery, depending on the precise execution of the virtual plan [9, 10]. Accurate and reproducible computer-guided surgery can allow for the flapless approach.

To fabricate the guide, however, there is necessary a series of complicated processes such as cone beam computed tomography (CBCT) imaging and acquisition, intraoral digital scan, alignment of CBCT and scan data, guide production with consideration for future prosthetic requirements, and implantation using implant drills consistent with the guide sleeve [2, 11]. Error can occur between the planned position before surgery and the actual implantation position as a result of the accumulation of all procedures including guide production, guide positioning, and guide movement. In addition, drill and mechanical errors could occur due to the offset of the guide and surgical instrument, and from the measurement and acquisition of the CT images [12–14]. Even if the guide was fabricated precisely, the guided surgery may be failed due to various reasons such as poor bone quality, thick mucosa, tissue inflation from local anesthesia, unstable fitness of the guide, or the presence of a bony dehiscence [1, 15]. In the instability case, therefore, the clinician should be changed the surgical plan to flap approach or freehand implant surgery. Although several factors affect the guided surgery systems such as image resolution of CBCT, image segmentation, field of view, type of tissue support, flap or flapless approach, and free- or full-guided implant placement [16, 17], there has been a lack of research that includes patients for whom guided surgery was not successful. Since guided surgery has become common in implant dentistry, it is important to emphasize its limitations, including the factors that indicate when guided surgery would be unsuitable [18].

The purpose of this study is to analyze the accuracy of computer-guided implant surgery based on superimposition of CBCT, which is widely used for this purpose [1], and discussed the limitations and future research of cases when implant placement differs from the planned surgical guide.

## Methods

This retrospective research was conducted under the approval of the Institutional Review Board of Seoul National University Bundang Hospital and independent ethics committees approved the protocol (IRB No. B-2009/637-101) with each participant providing written, informed consent. The study was performed according to the Declaration of Helsinki and the requirements of Good Clinical Practice.

All patients included in this study were adults who planned the computed-guided implant surgery according to the digital guide protocol based on CBCT (0.2-mm voxel size, Kodak 9500, Carestream Health, Inc., Trophy, France) at the Section of Dentistry of Seoul National University Bundang Hospital from December 2018 to January 2020. The inclusion criteria were as follows: (1) patients over 19 years of age; (2) CBCT data before and after implant placement; (3) informed consent from voluntary participants; (4) fabrication of the surgical guide according to the digital guide protocol, and (5) being partial edentulism (≤ 4 teeth loss in one arch). The exclusion criteria were as follows: (1) uncontrolled systemic disease or dentofacial-related syndrome; (2) being full edentulism; (3) related to pathologic conditions such periapical or periodontal abscesses, acute sinusitis, and untreated gingivitis or periodontitis.

### Computer-guided implant surgery process

**Fig. 1:**
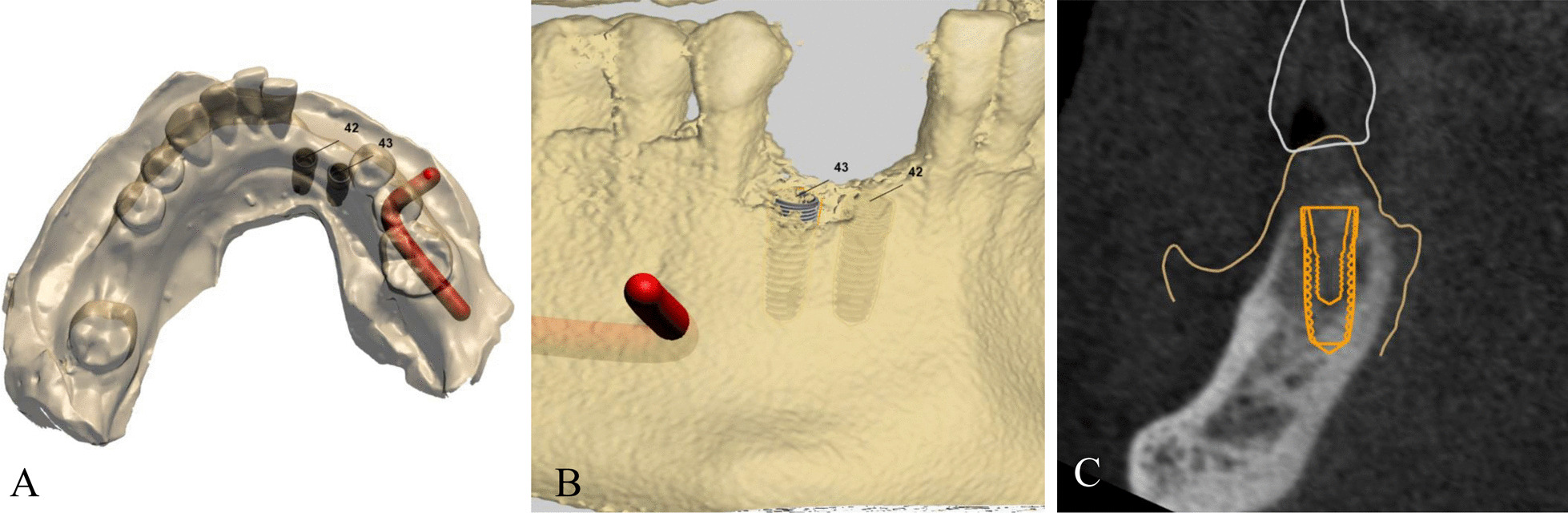
Superimposing the cone beam computed tomography and the intraoral scan. **A, B** Three-dimensional virtual images of the surgical site. **C **Virtual implant position planning with the relation of the implant to the surrounding bone structure

To establish an implant placement plan and create a digital implant guide, there was several steps to follow. First, we sent preoperative CBCT data. Second, a conventional gypsum material dental cast made from polyvinyl siloxane was scanned and imported to the Dentium Digital Center (Dentium, Suwon, Korea). Third. each model was scanned using Rainbow Scanner Prime (Dentium, Suwon, Korea) and superimposed with CBCT data based on the teeth. Fourth, a computer-guided implant plan was developed, including a digital wax-up of the edentulous region and accurate 3D location of the implants (Fig. 1). Based on the plan, finally, the tooth and mucosa supportive surgical guide was fabricated by using a stereolithography (SLA) 3D Printer (Dentium, Suwon, Korea). The manufactured guides were assessed for preoperative fitness in the oral cavity and adjusted as needed.

### Patient allocation according to surgical methods

According to the implant surgical procedure, implants that underwent placement according to a digital guide protocol based on the fabricated guide (VAROguide, Dentium, Suwon, Korea) were allocated in the experimental group, and those that could not be placed according to the guide protocol were allocated in the drop-out group. Among the experimental groups, the patients were classified the subgroups into flap and flapless approaches as groups 1 and 2, respectively. Surgery was performed by two expert surgeons (one oral and maxillofacial surgeon and one periodontal surgeon each; experience over 20 years in implant surgery, and over 5 years in the guide system).

### **Drop-out group: implants placed differently from the planned surgical guide**

**Fig. 2:**
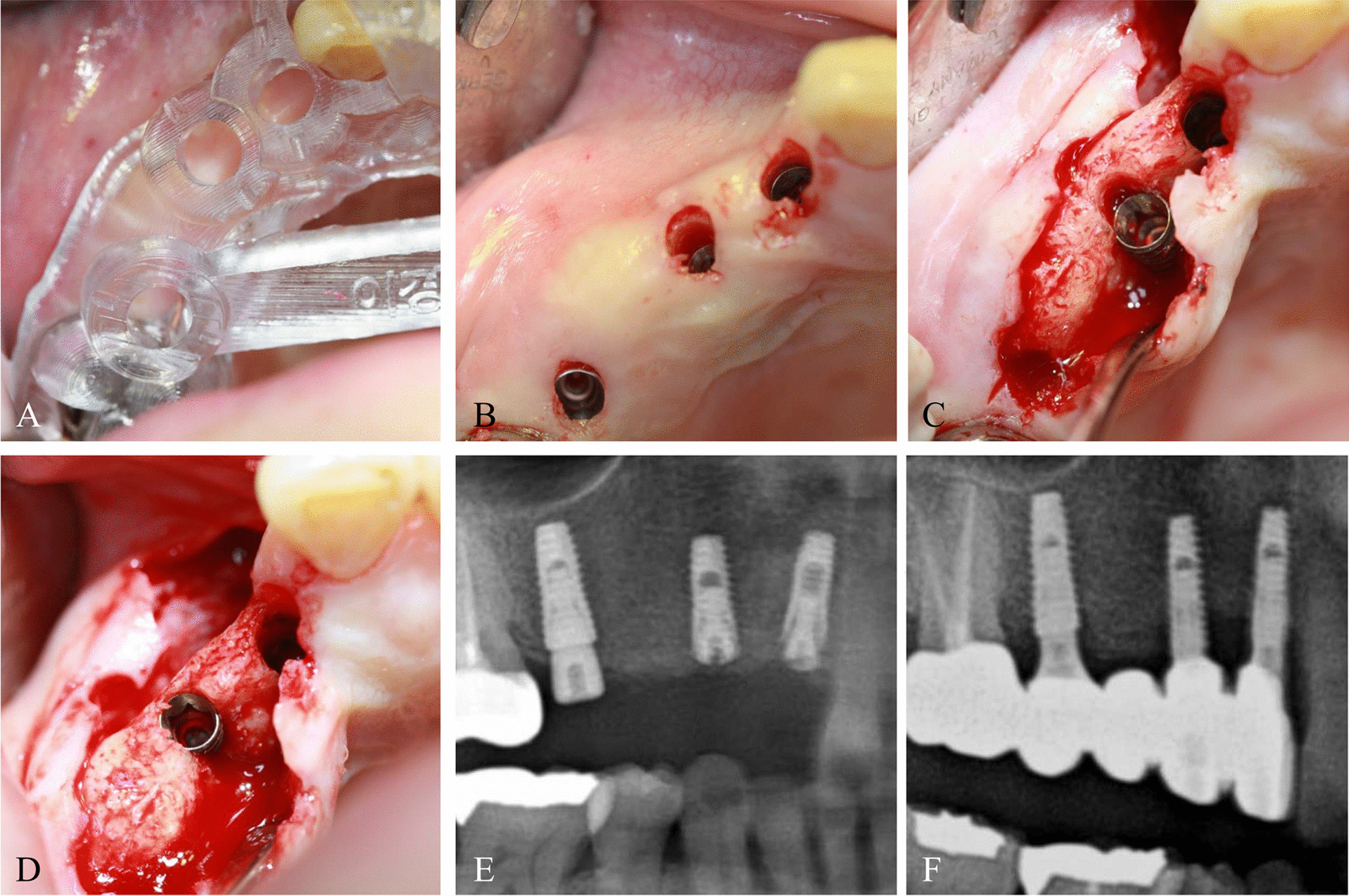
Representative intraoperative images in the drop-out group. **A** The fabricated guide positioned in the surgical site. **B** Guided implant placement through the flapless approach. **C** Since the primary stability of #13 was insufficient, the flap was elevated and a palatal bony dehiscence was observed. **D** The displaced implant fixture was removed and re-placed. The implant was located in the bony housing. **E** Postoperative radiography. **F** Prosthetic loading

Although the patients were planned implants based on preoperative CBCT images, some implants could not be accurately placed according to the fabricated guide, or were displaced compared with the planned position mainly due to poor bone quality and insufficient bone volume compared with the CBCT (Fig. 2).

### Group 1: implants placed from the fabricated guide according to the flapless approach

**Fig. 3:**
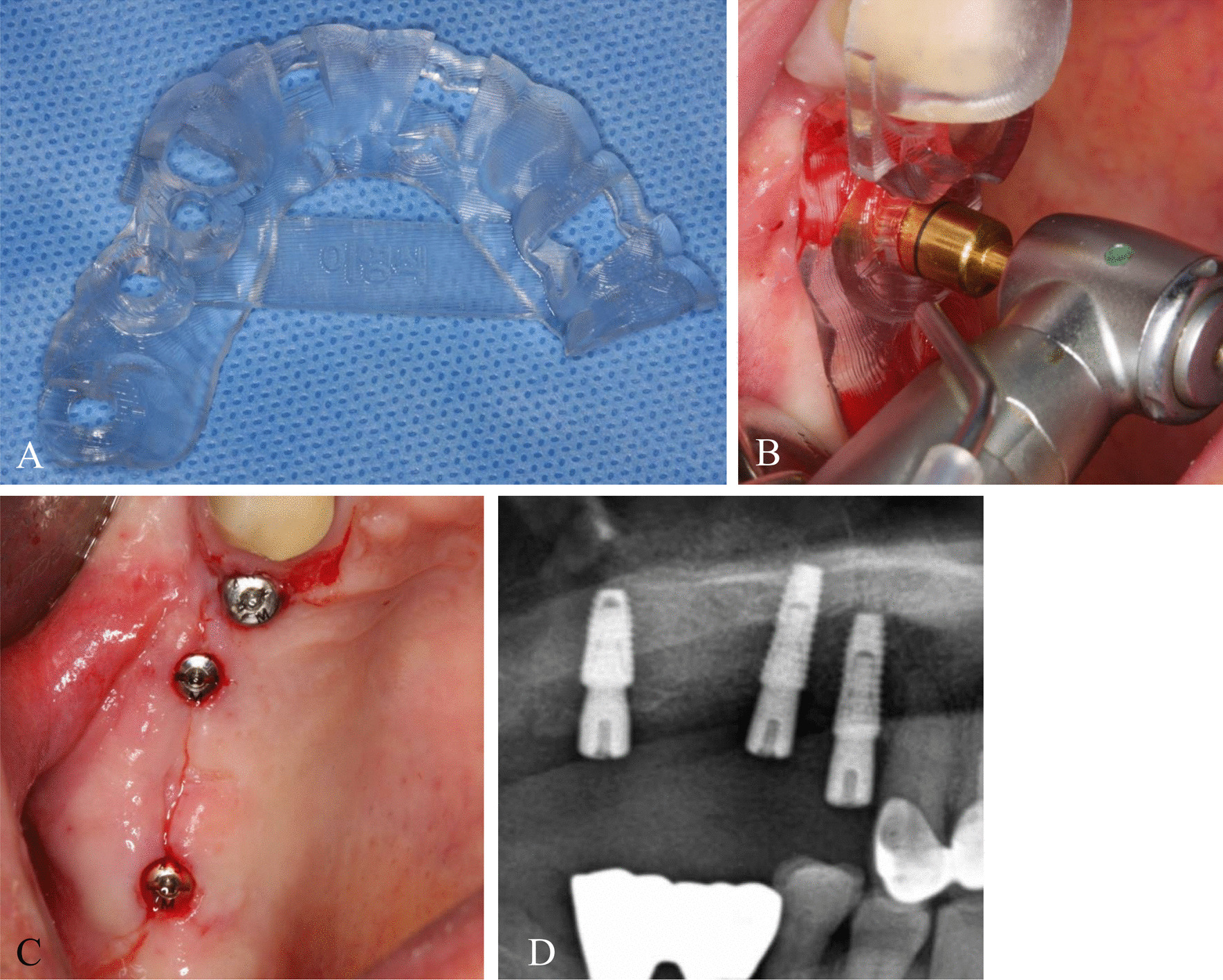
Guided implant placement with the surgical guide with the flapless approach. **A** Fabrication of the surgical guide for maxillary partial edentulism. **B** After the surgical guide was positioned in the surgical site, a 4.6-mm-diameter guided tissue punch was utilized for soft tissue removal with the indentation on the guide to stop drilling. **C** Following drilling by a 2.0-mm pilot-guided drill and a 4.2-mm tapered guided drill, the guided implant was placed in the planned location. **D** Postoperative radiography

A flapless technique was indicated if there was an appropriate amount of attached gingiva, sufficient bucco-lingual alveolar bone width, and residual bone to major anatomical structures, such as the maxillary sinus and inferior alveolar canal. After drilling according to the surgical guide through the guide sleeves, the implants were placed in the planned positions (Fig. 3). The diameter of the implant was chosen from preoperative planning, but the length varied slightly depending on the clinical circumstances.

### Group 2: implant placement from the fabricated guide according to the flapped approach

**Fig. 4:**
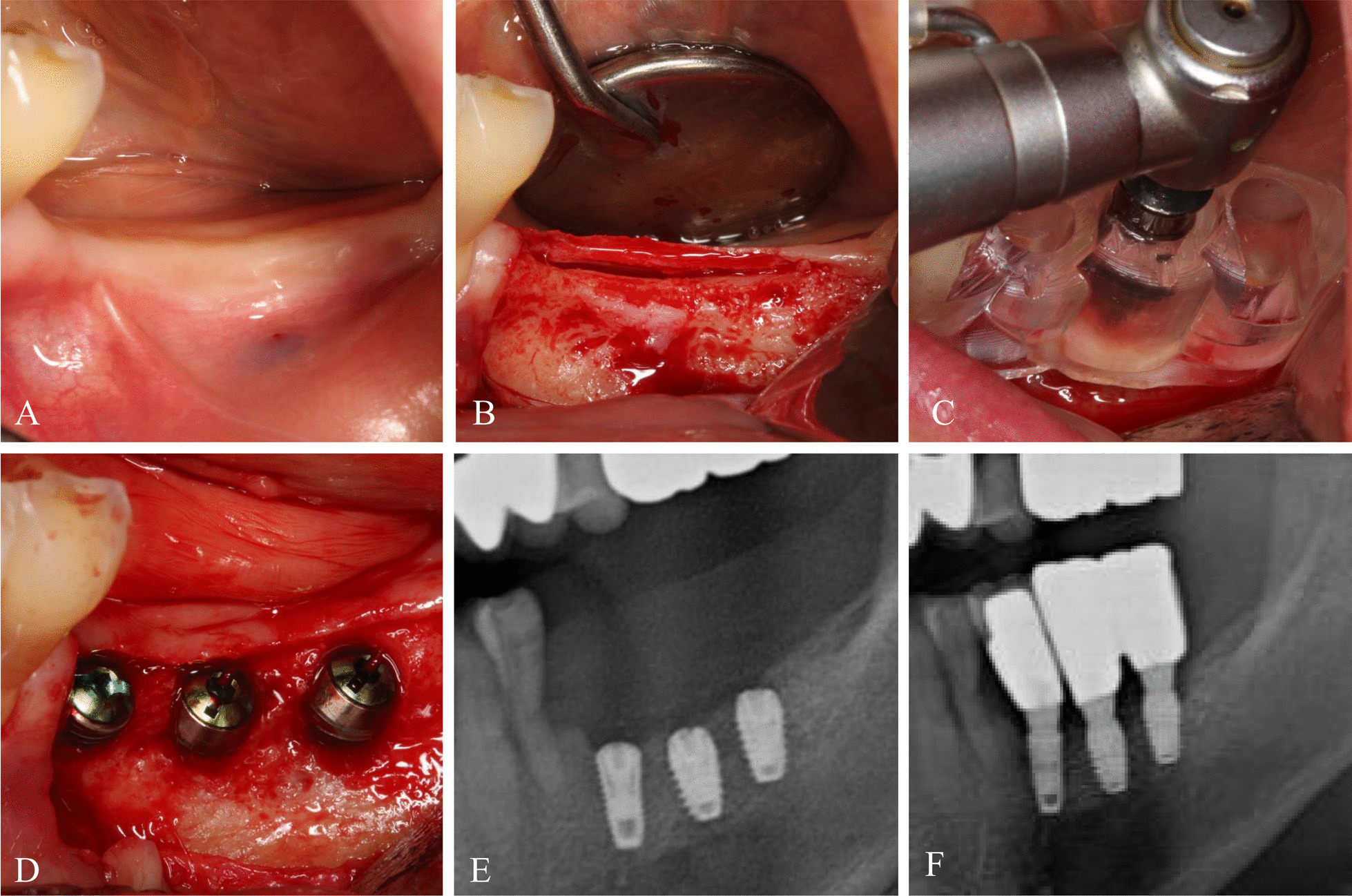
Guided implant placement with the surgical guide with the flapped approach. **A** Intraoral photograph of mandibular partial edentulism. **B** Since the horizontal alveolar bone dimension was insufficient, a mucoperiosteal full-thickness flap was elevated. **C** After the surgical guide was positioned in the surgical site, guided implant drilling was performed by a tapered guided drill used the drill stop indentation. **D** The guided implants were placed in the planned locations. **E** Postoperative radiography. **F** Prosthetic loading

The flapped approach was performed in cases where bone dimension was insufficient, bone augmentation was required, or primary stability was insufficient due to poor bone quality. After creating the incision, a full-thickness mucoperiosteal flap was elevated before fitting of the fabricated surgical guide. The implant was placed by fixation of the surgical guide with fingers. Drilling was performed sequentially according to the manufacturer’s guidelines (Dentium, Suwon, Korea), and the implants were placed until the indentation depth from the guide was reached (Fig. 4). However, the diameters and lengths of the drills were eventually changed depending on primary stability, bone quality, bone dimension, and the positions of the main anatomical structures. If necessary, bone augmentation was performed, and the wound was sutured after being covered with a shielding membrane. Depending on the degree of primary stability, submerged or non-submerged type implants were chosen.

### Postoperative care

All patients had postoperative CBCT immediately after the surgery, and were prescribed methylol cephalexin lysinate 500 mg bid for 5–7 days, celecoxib 200 mg bid for 5–7 days, and chlorhexidine (12% hexamedine solution 100 mL, BukwangPharm, Seoul, Korea) or benzydamine (15% Tantum gargle, 100 mL, SamaPharm, Seoul, Korea) oral gargling tid for 5–7 days. Two internal connection type implants with sandblasted, large grit, acid-etched surfaces were used: Superline (Dentium Co., Suwon, Korea) and Implantium (Dentium Co., Suwon, Korea).

### Measurements of the primary stability and evaluation of accuracy for the implant position

The primary stability was examined at fixture implantation, and implant stability quotients (ISQ) were measured with an Osstell Mentor device (Osstell, Gothenburg, Sweden) [19].

**Fig. 5:**
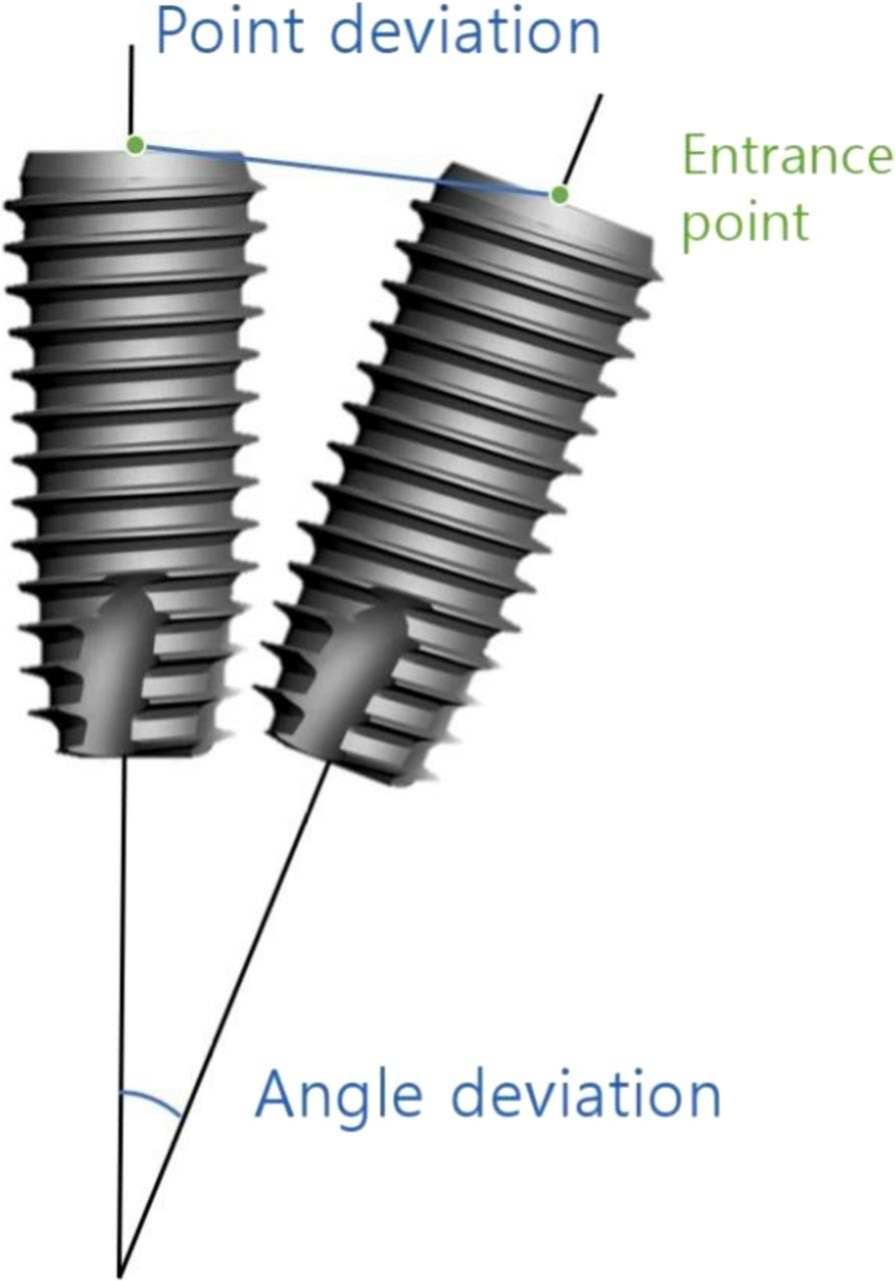
Measurement of deviations between the planned and placed positions of the implant

CBCT data were superimposed before and after the surgeries to evaluate the accuracy of implant positioning among the groups. Any changes in three-dimensional displacement and the angle of implant entrance after placement were evaluated by CBCT. To obtain images of standardized size, CBCT scans were performed at natural head positions using an occlusal plane aligner. A natural head position was obtained in an upright seated position and distanced gazing. CBCT data were extracted as a file of Digital Imaging and Communications in Medicine (DICOM). A 3-dimentional analysis program (OnDemand 3D, Cybermed, Seoul, Korea) was used to compare the preoperative planned location of the implant with the postoperative implant position by the automated registration software [20]. The software was superimposed on the basis of voxels’ gray level within the anterior cranial bases of the two CBCT. The anatomical structure of the anterior skull base was selected on the thalamus and ornamental and horizontal surfaces of the first input DICOM file. After automated superposition, the thalamus surface, tubular surface, and horizontal surface were formed in the tomographic direction of the initial input image [21]. To compare the position of the preoperative planned implant with the actual position of the implant after the operation, differences in the distance of the entry point and in the degree of the insertion angle were measured on the superimposed CBCT (Fig. 5).

SPSS statistics 25.0 (SPSS Inc., Chicago, IL, USA) program was used for statistical analysis, and significance was considered at a level of 0.05. The differences between the entrance displacement and the insertion angle of the planned and placed implants were measured to calculate the mean and standard deviation. Independent sample t-test was used to analyze the difference of the measurements among the groups.

## Results

A total of 89 implants (Dentium, Suwon, Korea) were placed in 34 patients (19 males and 15 females, average 62.1 years of age), and all implants were survived without failure during an average of 17.3 months within 0.5 mm marginal bone loss. Thirty-eight implants (42.7%) were placed without the fabricated guide, and 51 implants were allocated in the guided group. According to the groups, there were no significantly different on the age, sex, and the jaw (Table 1).

**Table 1:** Demographic information according to the groups

	Age (years)	P*	Sex (male:female)	P*	Jaw (Maxilla:Mandible)	P*
Drop-out group (N = 38)	65.8 ± 9.3	0.076^a^	19:19	0.784^a^	16:22	0.118^a^
Guided group (N = 51)	61.9 ± 10.8		27:24		30:21	
Group 1 (Flapped, N = 25)	59.7 ± 11.9	< 0.001^b^	13:12	0.895^b^	13:12	0.332^b^
Group 2 (Flapless, N = 26)	63.9 ± 9.4		14:12		17:9	

^*^Chi-Square tests

^a^Comparison between drop-out and guided groups

^b^Comparison between groups 1 and 2

**Fig. 6:**
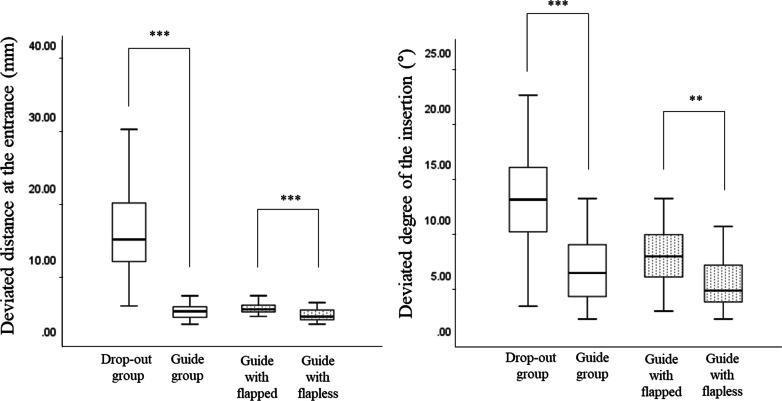
The results for the deviations between the planned and placed positions of the implant

Regarding the subgroup of the two guided groups, the flap (Group 1) and flapless approaches (Group 2) were performed for 26 and 25 implants, respectively (Table 2). The deviated position of the implant fixture was greater in group 1, compared with group 2 (Fig. 6). In particular, the implants in group 1 were more significantly deviated on the entrance displacement (3.18 ± 0.89 and 2.22 ± 0.88 mm, *P* < 0.001) compared with insertion degree (6.82 ± 2.71 and 4.27 ± 2.30°, *P* = 0.001).

**Table 2:** The position and primary stability of implants according to the groups

	Deviated distance at the entrance (mm)	P*	Deviated degree of the insertion (°)	P*	Primary stability (ISQ value)	P*
Drop-out group (N = 38)	14.53 ± 6.64	< 0.001^a^	11.75 ± 4.39	< 0.001^a^	66.49 ± 15.18	0.578 ^a^
Guided group (N = 51)	2.69 ± 1.00		5.52 ± 2.80		64.43 ± 18.26	
Group 1 (Flapped, N = 25)	3.18 ± 0.89	< 0.001^b^	6.82 ± 2.71	0.001^b^	55.84 ± 20.47	0.001 ^b^
Group 2 (Flapless, N = 26)	2.22 ± 0.88		4.27 ± 2.30		72.69 ± 10.97	

^*^Independent t-test

^a^Comparison between drop-out and guided groups

^b^Comparison between groups 1 and 2

**Fig. 7:**
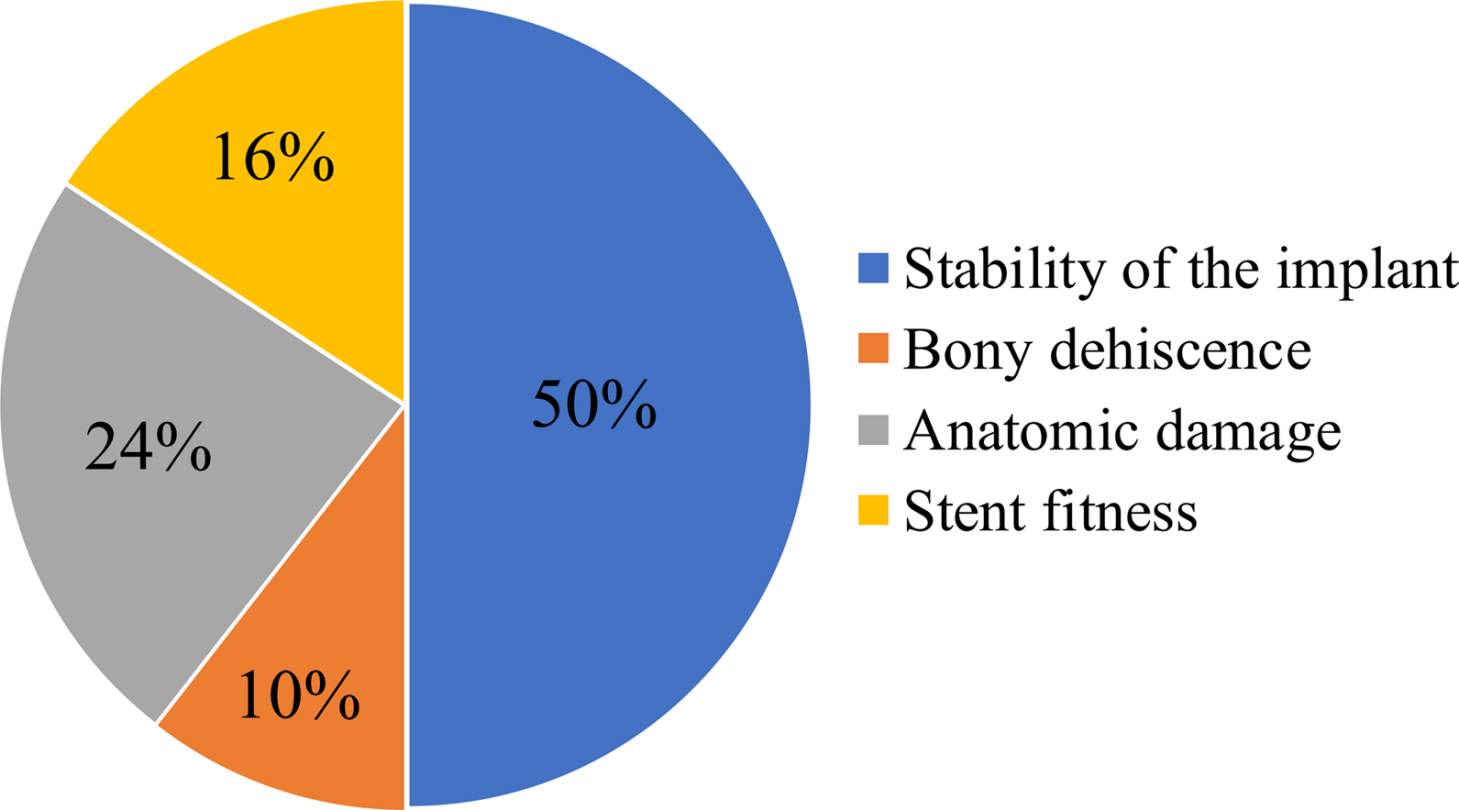
The causes preventing the use of the fabricated surgical guide among the 38 implants in the drop-out group

The deviated position of the implant fixtures was greater in the drop-out group compared to the guided group in terms of the displacement of the entrance point (14.53 ± 6.64 and 2.69 ± 1.00 mm, *P* < 0.001) and the degree of insertion (11.75 ± 4.39 and 5.52 ± 2.80°, *P* < 0.001) (Table 2). The primary stability was not significantly difference between the drop-out and guided groups (66.49 ±15.18 and 64.43 ± 18.26, *P* = 0.578). However, the flapped group was showed lower primary stability (55.84 ± 20.47) than the flapless group (72.69 ± 10.9, *P* = 0.001) Of the 38 implants in the drop-out group, the causes of changes in the plan were in the following order, insufficient primary stability (*n* = 19), risk of anatomic damage such as inferior alveolar canal proximity (*n* = 9), bone dehiscence during the drilling procedure (*n* = 4), and poor stent fitness (*n* = 6) (Fig. 7). Of the 19 cases of insufficient primary stability, 10 implants were placed deeper than the depth of implantation without changing the diameter or the length of the implant, while the others involved a change in diameter or length of the implant.

## Discussion

As a digital guided surgery has become very reproducible, many research were showed high accurate results to allow the deviation within 1.0 mm and 1.0° from guided position [22, 23]. However, errors could always occur between virtual planning and the surgical procedure. In 2018, the International Team for Implantology presented a consensus paper for the meta-analysis of the accuracy of guided implant surgeries, which showed a total mean error of 1.2 mm (1.0–1.4 mm) at the entry point and deviation of 3.5° (3.0°–4.0°), respectively [10]. They suggested that a safety margin of 2 mm should always be considered, because significant differences were reported in positions between the implant replicas in the plaster models created from CT data and the impressions taken from patients [24]. In addition, since the analysis program of this study was automatically superimposed based on the anterior skull base, the error could be more exaggerated in comparison to previous studies based on the adjacent structures of implants [1, 10, 12, 24–26]. Regarding the above mentioned considerations, the overall accuracy of the guide used this study was less accuracy for the reproducible implant placement through tooth-mucosa-supported surgical guides without fixation. However, the aim of this study is to discuss the limitation of the guided implant surgery based on the reason for drop-out group and factors affecting the error.

Computer-guided surgery cannot provide sufficient reproducibility for all implant patients, especially for those who have poor bone quality with irregular alveolar crests. In 2019, Al Yafi et al. demonstrated factors affecting the accuracy of guides including surgery-related factors such as flap design, reflection, and eccentric drilling through the sleeve while overcoming limited access to the posterior jaw or limited mouth opening [1]. Compared with the flapless approach, the flapped approach has disadvantages such as postoperative discomfort and increased surgical time. In 2017, a review concluded that the outcome of guided surgery with flapless approach indicated significantly more accuracy compared with flapped approach [27]. Some research was explained by the fact that positioning of the guide is more difficult because of the interference of the reflected flap [27, 28]. When considering guided surgery, the flap should be extensively reflected to prevent interference with the guide position and the possibility of reduced accuracy [29]. The accuracy with the flapped approach was significantly reduced in comparison with the flapless approach in this study, even though it was higher than freehand placement (Table 2).

In the case of unfavorable recipient bone, however, the flap approach has been necessary for intraoperative assessment to avoid several complications such as bone dehiscence, loss of implant stability, poor aesthetics, and invasion toward surrounding anatomic components [30–36]. Among the implants in this study, 60% of the drop-out implants failed to obtain primary stability within the bony housing along with the fabricated guide. Therefore, even though guided surgery has become very predictable with the flapless approach, it cannot be applied in some patients, especially in those who have poor bone quality where primary stability cannot be obtained or requiring bone grafting due to a narrow ridge [37].

Only few studies have been conducted on computer-guided implant placement in unfavorable bone requiring simultaneous augmentation with implant insertion. In 2021, Poli et al. reported five patients for computer-guided implant placements with computer-aided guided bone regeneration using tooth-supported surgical guides stabilized by the residual dentition. They exhibited accurate results on implant position deviation of 0.73 ± 0.21 mm and 3.05 ± 1.22° at the implant head and long-axis, respectively [37]. Since that research performed bone graft after securing the primary stability of implants, however, their results was reported in areas with sufficient bone quality even if the amount of bone is insufficient. However, the less primary stability of the flapped groups could be explained the less accuracy of the implant position in this study. To overcome this limitation, the guide should be allow the bone manipulation protocol for the poor bone quality, such as the bone expansion, splitting, compaction and underpreparation [38].

This study had several limitations such as heterogeneity to the surgeons and tooth position, and election bias due to retrospective design. Although guided surgery usually proceeds according to the postoperative plan, clinicians should not rely entirely on CBCT and the surgical guide, and be aware of possible complications such as offset of the guide or surgical instrument, poor guide fitness, and poor bone quality. In addition, further consideration should be conducted in cases that require a large amount of bone graft, or has close proximity to a major anatomic structure. Further research is necessary to maximize accuracy and suitability of the surgical guide even for the poor bone quality, which should be able to allow for predictable and reproducible implant position through the flap approach.

## Conclusions

Flapless guided implant surgery is more accurate than flap guided one in tooth-supported guides. However, poor bone quality and quantity can limit the applications of guided implant surgery. Future studies to improve implant treatment planning and guided surgery in cases with limited bone quality and quantity are needed.

## Data Availability

The datasets used and analyzed during the current study are available from the corresponding author upon reasonable request.

## Abbreviations

CBCT: Cone beam computed tomography
DICOM: Digital imaging and communications in medicine
SLA: Stereolithography

## References

[1] Al Yafi F, Camenisch B, Al-Sabbagh M (2019). Is digital guided implant surgery accurate and reliable?. Dent Clin N Am.

[2] D'Haese J, Ackhurst J, Wismeijer D, De Bruyn H, Tahmaseb A (2017). Current state of the art of computer-guided implant surgery. Periodontology 2000.

[3] Llamas-Monteagudo O, Girbés-Ballester P, Viña-Almunia J, Peñarrocha-Oltra D, Peñarrocha-Diago M (2017). Clinical parameters of implants placed in healed sites using flapped and flapless techniques: a systematic review. Med Oral Patol Oral Cir Bucal.

[4] Maier FM (2016). Initial crestal bone loss after implant placement with flapped or flapless surgery-a prospective cohort study. Int J Oral Maxillofac Implants.

[5] Yadav R, Agrawal KK, Rao J, Anwar M, Alvi HA, Singh K, Himanshu D (2018). Crestal bone loss under delayed loading of full thickness versus flapless surgically placed dental implants in controlled type 2 diabetic patients: a parallel group randomized clinical trial. J Prosthodont.

[6] Kumar D, Sivaram G, Shivakumar B, Kumar T (2018). Comparative evaluation of soft and hard tissue changes following endosseous implant placement using flap and flapless techniques in the posterior edentulous areas of the mandible-a randomized controlled trial. Oral Maxillofac Surg.

[7] Vohra F, Al-Kheraif AA, Almas K, Javed F (2015). Comparison of crestal bone loss around dental implants placed in healed sites using flapped and flapless techniques: a systematic review. J Periodontol.

[8] Llamas-Monteagudo O, Girbés-Ballester P, Viña-Almunia J, Peñarrocha-Oltra D, Peñarrocha-Diago M (2017). Clinical parameters of implants placed in healed sites using flapped and flapless techniques: a systematic review. Medicina oral, patologia oral y cirugia bucal.

[9] Gargallo-Albiol J, Barootchi S, Salomó-Coll O, Wang HL (2019). Advantages and disadvantages of implant navigation surgery. A systematic review. Ann anat Anat Anz Off Organ Anat Ges.

[10] Tahmaseb A, Wu V, Wismeijer D, Coucke W, Evans C (2018). The accuracy of static computer-aided implant surgery: a systematic review and meta-analysis. Clin Oral Implant Res.

[11] Jung-Kyo L, Yeo-Gab K (2011). An anatomical study on the mandibular medial surface by CBCT analysis for safer implant placement. JKAOMS.

[12] Vercruyssen M, Cox C, Coucke W, Naert I, Jacobs R, Quirynen M (2014). A randomized clinical trial comparing guided implant surgery (bone- or mucosa-supported) with mental navigation or the use of a pilot-drill template. J Clin Periodontol.

[13] Tatakis DN, Chien H-H, Parashis AO (2019). Guided implant surgery risks and their prevention. Periodontology 2000.

[14] Flügge T, Derksen W, Te Poel J, Hassan B, Nelson K, Wismeijer D (2017). Registration of cone beam computed tomography data and intraoral surface scans—a prerequisite for guided implant surgery with CAD/CAM drilling guides. Clin Oral Implant Res.

[15] Behneke A, Burwinkel M, Behneke N (2012). Factors influencing transfer accuracy of cone beam CT-derived template-based implant placement. Clin Oral Implant Res.

[16] Jacobs R, Salmon B, Codari M, Hassan B, Bornstein MM (2018). Cone beam computed tomography in implant dentistry: recommendations for clinical use. BMC Oral Health.

[17] Hassan B, Couto Souza P, Jacobs R, de Azambuja BS, van der Stelt P (2010). Influence of scanning and reconstruction parameters on quality of three-dimensional surface models of the dental arches from cone beam computed tomography. Clin Oral Investig.

[18] Unsal GS, Turkyilmaz I, Lakhia S (2020). Advantages and limitations of implant surgery with CAD/CAM surgical guides: a literature review. J Clin Exp Dent.

[19] Sargolzaie N, Samizade S, Arab H, Ghanbari H, Khodadadifard L, Khajavi A (2019). The evaluation of implant stability measured by resonance frequency analysis in different bone types. JKAOMS.

[20] Ku JK, Choi SK, Lee JG, Yu HC, Kim SY, Kim YK, Shin Y, Lee NK (2021). Analysis of sagittal position changes of the condyle after mandibular setback surgery across the four different types of plating systems. J Craniofac Surg.

[21] Park JY, Bae SY, Lee JJ, Kim JH, Kim HY, Kim WC (2017). Evaluation of the marginal and internal gaps of three different dental prostheses: comparison of the silicone replica technique and three-dimensional superimposition analysis. J Adv Prosthodont.

[22] Bencharit S, Staffen A, Yeung M, Whitley D, Laskin DM, Deeb GR (2018). In vivo tooth-supported implant surgical guides fabricated with desktop stereolithographic printers: fully guided surgery is more accurate than partially guided surgery. J Oral Maxillofac Surg.

[23] Suriyan N, Sarinnaphakorn L, Deeb GR, Bencharit S (2019). Trephination-based, guided surgical implant placement: a clinical study. J Prosthet Dent.

[24] Komiyama A, Pettersson A, Hultin M, Näsström K, Klinge B (2011). Virtually planned and template-guided implant surgery: an experimental model matching approach. Clin Oral Implant Res.

[25] Orentlicher G, Horowitz A, Kobren L (2019). Computer-guided dental implant treatment of complete arch restoration of edentulous and terminal dentition patients. Oral Maxillofac Surg Clin N Am.

[26] D'Haese J, Van De Velde T, Komiyama A, Hultin M, De Bruyn H (2012). Accuracy and complications using computer-designed stereolithographic surgical guides for oral rehabilitation by means of dental implants: a review of the literature. Clin Implant Dent Relat Res.

[27] Zhou W, Liu Z, Song L, Kuo C-L, Shafer DM (2018). Clinical factors affecting the accuracy of guided implant surgery—a systematic review and meta-analysis. J Evid Based Dent Pract.

[28] Vieira DM, Sotto-Maior BS, de Souza Barros CAV, Reis ES, Francischone CE (2013). Clinical accuracy of flapless computer-guided surgery for implant placement in edentulous arches. Int J Oral Maxillofac Implants.

[29] Lal K, White GS, Morea DN, Wright RF (2006). Use of stereolithographic templates for surgical and prosthodontic implant planning and placement. Part I. The concept. J Prosthodont.

[30] Rosenfeld AL, Mandelaris GA, Tardieu PB (2006). Prosthetically directed implant placement using computer software to ensure precise placement and predictable prosthetic outcomes. Part 1: diagnostics, imaging, and collaborative accountability. Int J Periodontics Restor Dent.

[31] Abboud M, Wahl G, Calvo-Guirado JL, Orentlicher G (2012). Application and success of two stereolithographic surgical guide systems for implant placement with immediate loading. Int J Oral Maxillofac Implants.

[32] Jin-Hong K, Hee-Keun P, Moon-Key K, Sang-Hoon K (2012). Life-threatening airway obstruction after flapless implant placement in the anterior mandible. JKAOMS.

[33] Apostolakis D, Brown JE (2012). The anterior loop of the inferior alveolar nerve: prevalence, measurement of its length and a recommendation for interforaminal implant installation based on cone beam CT imaging. Clin Oral Implant Res.

[34] Van de Velde T, Glor F, De Bruyn H (2008). A model study on flapless implant placement by clinicians with a different experience level in implant surgery. Clin Oral Implant Res.

[35] Better H, Chaushu L, Nissan J, Xavier S, Tallarico M, Chaushu G (2018). The feasibility of flapless approach to sinus augmentation using an implant device designed according to residual alveolar ridge height. Int J Periodontics Restor Dent.

[36] Galindo-Moreno P, Padial-Molina M, Avila G, Rios HF, Hernández-Cortés P, Wang HL (2012). Complications associated with implant migration into the maxillary sinus cavity. Clin Oral Implant Res.

[37] Poli PP, Muktadar AK, Souza FÁ, Maiorana C, Beretta M (2021). Computer-guided implant placement associated with computer-aided bone regeneration in the treatment of atrophied partially edentulous alveolar ridges: a proof-of-concept study. J Dent Sci.

[38] Mittal Y, Jindal G, Garg S (2016). Bone manipulation procedures in dental implants. Indian J Dent.

